# Intravenous self-administration of delta-9-THC in adolescent rats produces long-lasting alterations in behavior and receptor protein expression

**DOI:** 10.1007/s00213-020-05684-9

**Published:** 2020-10-27

**Authors:** Sierra J. Stringfield, Mary M. Torregrossa

**Affiliations:** 1 Department of Psychiatry, University of Pittsburgh, 450 Technology Drive, Pittsburgh, PA 15219, USA; 2 Center for Neuroscience, University of Pittsburgh, 4200 Fifth Ave, Pittsburgh, PA 15213, USA

**Keywords:** Adolescent, Δ-9-tetrahydrocannabinol, self-administration, cannabinoid, sex-differences, GABA, glutamate, reinstatement, prefrontal cortex

## Abstract

**Rationale:**

Initial exposure to cannabinoids, including Δ-9-tetrahydrocannabinol (THC), often occurs during adolescence. Considerable neurodevelopmental alterations occur throughout adolescence, and the environmental insult posed by exogenous cannabinoid exposure may alter natural developmental trajectories. Multiple studies suggest that long-lasting deficits in cognitive function occur as a result of adolescent cannabis use, but considerable variability exists in the magnitude of these effects.

**Objectives:**

We sought to establish a novel procedure for achieving intravenous THC self-administration in adolescent rats in order to determine if volitional THC intake in adolescence produced indices of addiction-related behavior, altered working memory performance in adulthood, or altered the expression of proteins associated with these behaviors across several brain regions.

**Methods:**

Male and female adolescent rats learned to operantly self-administer escalating doses of THC intravenously from PD 32–51. Upon reaching adulthood they were tested in abstinence for cued reinstatement of THC-seeking and working memory performance on a delayed-match-to-sample task. In a separate cohort, glutamatergic, GABAergic, and cannabinoid receptor protein expression was measured in multiple brain regions.

**Results:**

Both male and female adolescents self-administered THC and exhibited cue-induced lever pressing throughout abstinence. THC exposed males exhibited slightly enhanced working memory performance in adulthood, and better performance positively correlated with total THC self-administered during adolescence. Adolescent THC-exposed rats exhibited reductions in CB1, GABA and glutamate receptor protein, primarily in the prefrontal cortex, dorsal hippocampus, and ventral tegmental area.

**Conclusions:**

These results suggest that THC exposure at self-administered doses can produce moderate behavioral and molecular alterations, including sex-dependent effects on working memory performance in adulthood.

## Introduction

Cannabis is the most widely used illicit substance among adolescents in the United States, and initiation of cannabis use often occurs during adolescence (SAMHSA 2019; Johnston et al. 2020). The growing trend toward legalization of cannabis for recreational or medical use, combined with a reduced perception of harm among adolescents (SAMHSA 2019), presents a need for greater understanding of the acute and longitudinal effects of cannabinoid exposure. Adolescence is a time of marked synaptic and neuronal development, and exposure to multiple drugs of abuse during this period can produce prolonged deficits in cognitive functioning (Spear 2016; Mooney-Leber and Gould 2018). The endocannabinoid system is developing during this time, while also contributing to the significant neuroplastic and neurochemical reorganization occurring in key neurotransmitter systems and brain regions (Sturman and Moghaddam 2011). Both human and animal studies indicate that the environmental insult from exposure to psychoactive cannabinoids may cause persistent changes to the maturation of the endocannabinoid system as well as other brain circuits involved in cognitive, emotional, and social processing (Broyd et al. 2016; Rubino and Parolaro 2016). Cannabinoid exposure has been associated with increased risk for psychiatric diseases (Chadwick et al. 2013), psychotic-like symptoms (Bossong and Niesink 2010), and cognitive deficits that persist into adulthood (Meier et al. 2012). However, due to variability in populations, dose, length of exposure, and confounds such as exposure to other substances, the specific long-term effects of cannabinoids are not clear (Ganzer et al. 2016; Scott et al. 2018).

Animal models provide the opportunity to perform controlled studies of the consequences of exposure to a specific drug. Multiple animal models of drug delivery have been utilized to administer behaviorally relevant doses of cannabinoids to adolescent animals. Current models include vapor/smoke inhalation (both passive and self-administered), oral self-ingestion, and experimenter-administered injections to provide information about the cognitive or cellular alterations caused by exposure to synthetic or plant-derived cannabinoids. These rodent studies have demonstrated long-lasting alterations in salience processing, deficits in spatial or object recognition memory , depression- and anxiety-like phenotypes, and increased drug-seeking in adulthood (O’Shea et al. 2004; Ellgren et al. 2007; Rubino et al. 2008; Rubino et al. 2009a; Abush and Akirav 2012; Stopponi et al. 2014; Scherma et al. 2016; Renard et al. 2017; Schoch et al. 2018; Kruse et al. 2019). Conversely, and similar to human studies, adolescent exposure to cannabis or THC smoke is also reported to produce little to no detrimental effects in adulthood (Bruijnzeel et al. 2019). The behavioral alterations produced by cannabinoid exposure in animal models are supported by synaptic and neuronal changes in key brain regions that develop during adolescence such as the prefrontal cortex, hippocampus, and striatum. Adolescent cannabinoid exposure has been associated with long-lasting modifications to synaptic plasticity and cell firing patterns in the prefrontal cortex and hippocampus (Rubino et al. 2009b; Raver et al. 2013; Miller et al. 2019), and GABAergic and glutamatergic transmission throughout the brain (Cass et al. 2014; Zamberletti et al. 2014; Lovelace et al. 2015; Rubino et al. 2015; Renard et al. 2018). In human cannabis users, postmortem examination of cannabinoid receptors revealed reduced CB1 receptor expression and binding in the caudate and accumbens (Villares 2007). CB1 receptors vary in expression throughout development (Fernández-Ruiz et al. 2000; Mato et al. 2003) and adolescent exposure by multiple routes of administration can cause acute (Burston et al. 2010; Silva et al. 2015) and long-lasting (Kruse et al. 2019) downregulation or desensitization of CB1 receptors.

In studies that include both males and females, sex differences in the effects of adolescent cannabinoid exposure have also been reported (Rubino and Parolaro 2015). For example adolescents and adults show sex differences in tolerance to the physiological effects of THC (Wakley et al. 2014; Nguyen et al. 2020), and cognitive and addiction-associated behavioral effects of adolescent exposure may emerge only in females (Biscaia et al. 2008; Higuera-Matas et al. 2008; Rubino et al. 2008; Rubino et al. 2009a), or males (Kruse et al. 2019). On the biochemical level, fluctuations in transcription factors, protein expression, and synaptic transmission may also be sex-specific (Rubino et al. 2008; Burston et al. 2010; Castelli et al. 2014; Poulia et al. 2019; Kruse et al. 2019).

The well-characterized exposure models of passive or experimenter-administered cannabinoids are difficult to adapt to addiction-associated questions such as relapse, that are better modeled using tests of reinstatement. In adult animals, recent advancements in operant models of THC and cannabinoid self-administration provide a method for investigating these questions. Although self-administration of the synthetic cannabinoids WIN 55215–2 or CP55,940 can be achieved in both adolescent and adult animals (Braida et al. 2001; Fattore et al. 2001; Lefever et al. 2014; Kirschmann et al. 2017b; Kirschmann et al. 2017a) self-administration of THC has been notoriously difficult to achieve in rodents (Takahashi and Singer 1979; Justinova et al. 2005). Recently, novel developments in adult operant vapor inhalation (Freels et al. 2020) and intravenous self-administration (Wakeford et al. 2017; Spencer et al. 2018) have achieved exposure to physiologically relevant doses of THC and resulted in acute and chronic alterations to addiction-associated brain regions and neuronal plasticity. We have previously shown that intravenous self-administration of synthetic cannabinoids in adolescents actually results in improved working memory performance in adulthood, though also induces relapse-like behavior, as well as associated changes in synaptic development (Kirschmann et al. 2017a; Kirschmann et al. 2017b).

Given the conflicts within the literature concerning the long-term effects of adolescent cannabinoid exposure, where human studies suggest a moderate effect and animal models vary based on dose and route of exposure, there is a need to increase researchers ability to perform studies using THC self-administration models. Self-administration allows the direct investigation of behavioral questions that arise from giving control of dose and timing to the animal, such as questions of individual differences in preferred intake and sex-differences in protracted response to reinforcing doses of the drug. In this study, we establish a model of THC self-administration in adolescent male and female Sprague Dawley rats. We investigate long-term effects on receptor protein expression in multiple addiction-associated brain regions that are particularly vulnerable to adolescent drug exposure and identify potential sex-differences in relapse-like behavior and cognitive function measured in adulthood.

## Methods

### Animals

Male (n=50) and female (n=53) Sprague Dawley rats (Envigo, Indianapolis, IN) arrived on postnatal day (PD) 22 and were housed in a temperature and humidity controlled room on a 12:12 h light:dark cycle. All behavioral experiments were conducted during the light cycle. Rats were pair housed prior to surgery and had access to food and water *ad libitum* unless otherwise noted. All procedures were approved by the University of Pittsburgh Institutional Animal Care and Use Committee and were performed in accordance with the National Institutes of Health *Guide for the Care and Use of Laboratory Animals*.

### Drugs

Δ-9-Tetrahydrocannabinol (THC) was provided by the National Institute on Drug Abuse’s Drug Supply program. Stock solutions were prepared by adding 100–200μl Tween 80 to an aliquot of THC solution before the ethanol was evaporated off using a steady stream of nitrogen gas. The stock solution was then brought to a volume of 1mL with sterile 0.9% saline. Working solutions were prepared immediately prior to each self-administration session by diluting the stock solutions with additional saline to reach the desired concentration of THC, with a final concentration of Tween 80 between 0.05–0.1%. Vehicle solutions were made from Tween 80 with an equivalent volume of ethanol evaporated, and dilution in saline.

### Surgery

All rats were implanted with intravenous jugular catheters as described previously (Rich et al. 2016). Rats (PD 25–26) were anesthetized with ketamine (87.5mg/kg) and xylazine (5mg/kg) and given Rimadyl (5mg/kg, Carprofen; Zoetis, Kalamazoo, MI) as an analgesic. All rats were single housed and given 6–7 days to recover from surgery before behavioral testing. Gentamicin (5mg/ml) and heparin (30USP/ml) in sterile saline were infused daily to maintain catheter patency.

### Intravenous THC Self-Administration, Lever Extinction, and Reinstatement

During self-administration, adolescent rats (PD 32–51) were food restricted to 13–18g rat chow per day. Rats were trained to lever press for THC in standard operant conditioning chambers (Med Associates, St Albans, VT) during daily 2-hour sessions. Each chamber contained two retractable levers with stimulus lights directly above each lever, house light, food magazine, tone generator, and fan. Rats acquired THC self-administration by lever pressing for escalating doses of the drug on a fixed ratio (FR) 1 schedule of reinforcement over the course of 9 days, before maintaining the final concentration of drug for 11 days. All rats in THC groups initially self-administered a dose of 3 μg/kg/infusion for days 1–3, before escalating to 10 μg/kg/infusion for days 4–6, and 30 μg/kg/infusion for days 7–9. One cohort of animals remained at the 30 μg/kg dose for the remainder of self-administration (days 10–20) while a separate cohort escalated to 100 μg/kg/infusion for these days. Rats were randomly assigned to an active lever, and responses on the active lever resulted in an infusion of THC or an equivalent volume of vehicle paired with presentation of a 10 s light and tone compound conditioned stimulus followed by a 10 s timeout. Responses on the inactive lever were recorded but had no programmed consequences.

After self-administration, rats completed 9 days of 1-hour lever extinction sessions (PD 52–60). Nine days of extinction was chosen to allow sufficient time for drug washout (Gunasekaran et al. 2009). Rats were placed in the self-administration chamber where active and inactive levers were available, but lever pressing did not result in drug delivery or cue presentation. Extinction criteria were set at fewer than 20 active or inactive lever presses by the final day of extinction. On abstinence days 10 and 30 (PD 61 and 81), rats completed a 30 min cued reinstatement session. Rats were again placed into the self-administration chamber and pressing on the active lever resulted in a 5 s presentation of the conditioned stimulus without drug or vehicle infusion.

### Hot Water Tail Withdrawal

On days 19 or 20 of self-administration, a subset of THC and vehicle animals were tested for antinociception using the hot water tail withdrawal assay. Immediately prior to the self-administration session and immediately after, each rat was held by the experimenter and 4 inches of the tail was submerged into a 50°C water bath. The time required to remove the tail from the bath was recorded.

### Delayed-Match-to-Sample Working Memory Task

At PD 63, rats that had previously completed self-administration and lever extinction began training on the delayed-match-to-sample working memory task (as previously described, Kirschmann et al., 2017) during daily 1-hour sessions. Rats were trained in operant chambers (Med Associates) equipped with a panel containing 5 illuminated nose poke apertures and a magazine, sucrose pellet dispenser, and house light on the opposite wall. Rats were initially trained to respond into any illuminated aperture on a FR1 schedule to receive a sucrose pellet reward. Next, animals were trained to respond in a specific illuminated aperture. After completing this phase of training, animals were then introduced to increasingly difficult delay periods ranging from 0.5 – 24 s. Rats learned to respond in a specific illuminated aperture during a sample phase which was followed by a 0.5 s delay before initiation of the choice phase, where the originally sampled aperture was illuminated along with 2 directly adjacent apertures. For example, if the sample aperture was in the center of the panel, the apertures to the left and right would be illuminated. If the sample aperture was on the edge of the panel the two apertures to the left or right would be illuminated. The animal had to choose the originally sampled aperture to receive a sucrose pellet reward. Once rats learned to respond correctly (80% correct trials), increasingly difficult delays were introduced. Rats responded for sucrose pellet reinforcers in blocks of trials where 7 delays (ranging from 0.5 – 6 s) would elapse between sample and choice phases. Each delay was presented in a random order, and all delays would occur in a block before repeating. After maintaining 80% correct trials at the 0.5 s delay, rats advanced to a longer range of delay periods (0.5 – 12 s). A final test session was conducted during the first day of training on a 0.5 – 24 s delay set. The number of correct and incorrect trials at each delay were recorded for each session.

### Western Immunoblot

A separate group of adolescent male and female rats were trained to self-administer vehicle or THC using the procedure described above and reached a maximum dose of 100 μg/kg/infusion. These animals then remained in the home cage until PD 90, corresponding to the approximate age of working memory testing, and were euthanized by rapid decapitation. Brains were flash-frozen in isopentane on dry ice and stored at −80°C until further processing. Brains were sliced into 1mm coronal sections and the prelimbic (PrL) and infralimbic (IL) cortices, nucleus accumbens (NAc), basolateral amygdala (BLA), dorsal hippocampus (DH) and ventral tegmental area (VTA) were dissected using a 1mm tissue punch. Regions of interest were fractionated into soluble and membrane bound components (Bañuelos et al. 2014; Kirschmann et al. 2017b). Protein concentration was determined using a bicinchoninic acid assay (BCA Protein Assay, Thermo-Scientific Pierce, Waltham, MA). Tissue samples were then reduced in Laemmli sample buffer (Bio-Rad, Hercules, CA) and boiled at 90°C for 5 minutes. 20μg protein was loaded onto 4–20% Tris-glycine gels (Invitrogen, Carlsbad, CA) and separated by SDS-PAGE. Separated proteins were then transferred to polyvinylidene fluoride (PVDF) membranes and incubated at room temperature for 1 hour in blocking solution (5% non-fat dry milk in PBS containing 0.1% Tween 20). Membranes were then incubated overnight at 4°C in primary antibodies against proteins of interest: GABA receptor subunits GABA_A_R1_α_, (1:10000; Abcam, Cambridge, UK) and GABA_B_R2 (1:1000; Cell Signaling, Danvers, MA); GluR2/3 (1:1000; MilliporeSigma), CB1 (1:500; Alomone Labs, Jerusalem, Israel) and GAPDH loading control (1:1000; MilliporeSigma). Membranes were then incubated with secondary fluorescent antibodies (IRDye 800 CW anti-rabbit, 1:5000; IRDye 680 CW anti-mouse, 1:5000) at room temperature. All antibodies were diluted in 1:1 Li-COR Odyssey blocking buffer (Li-COR, Lincoln, NE) and PBS. Membranes were imaged using a Li-COR Odyssey imaging system and analyzed with Li-COR Image Studio software. Each protein sample was normalized to its respective GAPDH loading control and normalized to within gel vehicle controls.

### Statistical Analysis

Statistical analyses were conducted using GraphPad Prism version 8.2 (GraphPad, San Diego, CA) and STATISTICA (TIBCO, Palo Alto, CA). Lever presses, infusions, discrimination indices, tail withdrawal latencies, and accuracy on the working memory task were analyzed by 2- or 3-way repeated measures ANOVA (α=0.05) followed by Bonferroni post-hoc comparisons when appropriate. Mean differences during self-administration were analyzed by paired t-test. Discrimination indices during self-administration were calculated using the formula [(Active Lever- Inactive Lever) / Total Lever Presses]. Protein expression from Western blots were analyzed by unpaired t-tests.

## Results

### Adolescent rats self-administer escalating doses of THC

**Fig. 1:**
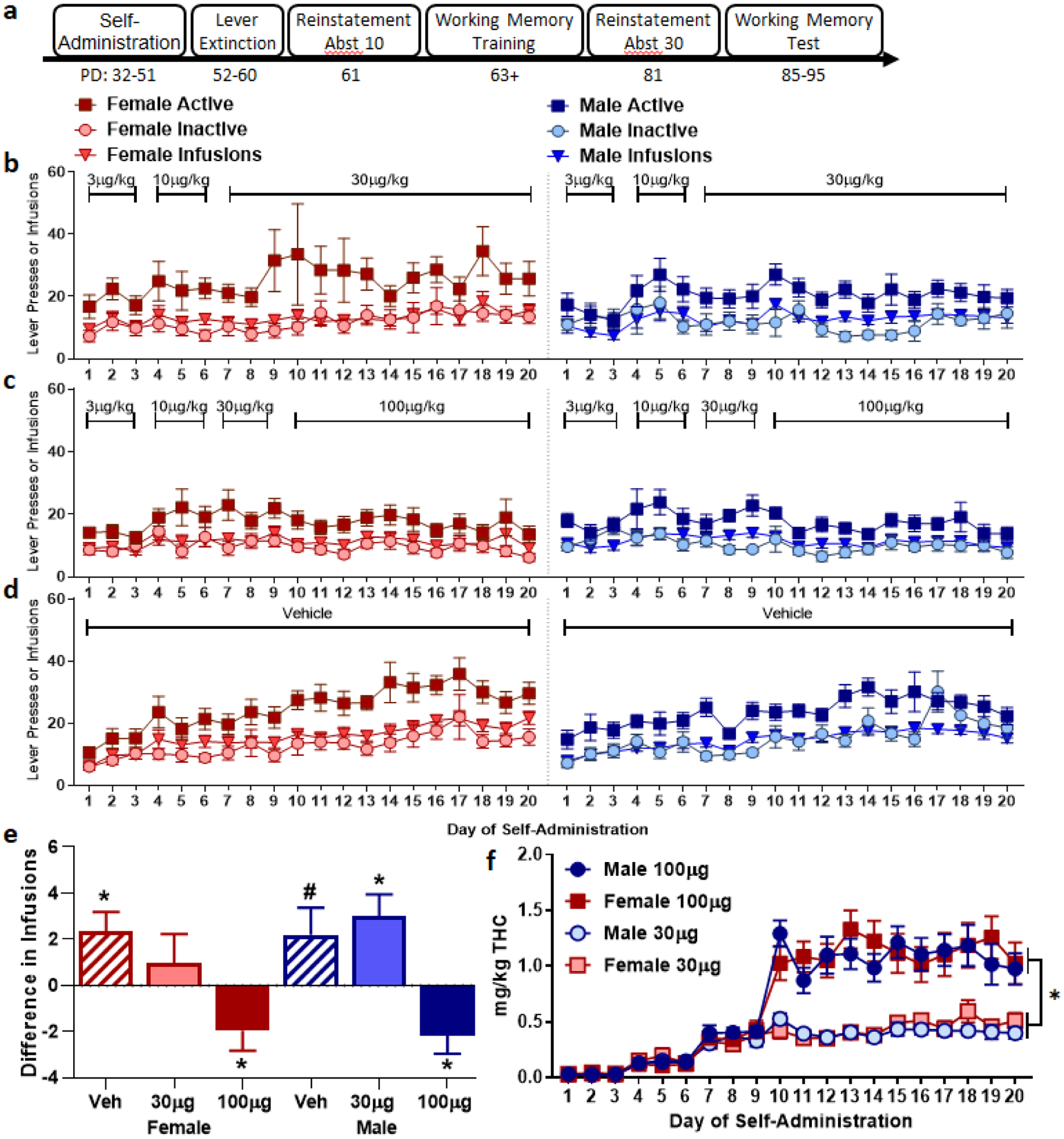
THC self-administration in adolescent male and female rats. (a) Timeline of behavioral experiments. (b-d) Active and inactive lever presses or infusions across 20 days of self-administration, separated by sex to facilitate visualization. (b) Self-administration by adolescent rats taking the 30 μg/kg/infusion dose of THC, female (left, n=14) or male (right, n=12). (c) Adolescent rats self-administering the 100 μg/kg/infusion dose, (n=22 female, n=22 male). (d) Adolescent rats self-administering vehicle (n=17 female, n=16 male). (e) Male and female adolescent rats reduced the number of infusions received upon escalating to the 100 μg/kg/infusion dose of THC while all other groups increased or did not change, difference between self-administration days 7–9 and days 10–12 * p<0.05, # p<0.1. (f) Daily intake in males and females self-administering the 30 μg/kg or 100 μg/kg doses of THC, * main effect of dose, p<0.05

Adolescent male and female rats were trained to self-administer escalating doses of THC or Vehicle (n=12–22 per group) throughout 20 days of adolescence (PD 32–51, Fig 1b–d). A 3-way repeated measures ANOVA with day of self-administration as a within subjects factor and sex and THC dose [0 (vehicle), 30, 100 μg/kg/infusion] as between-subject factors revealed a significant main effect of dose on active lever pressing [F_(2,99)_ = 6.44, p<0.01], a main effect of day [F_(19,1881)_ = 4.3, p<0.001], and a significant day х dose interaction [F_(38,1881)_ = 2.32, p<0.001]. Analysis of this interaction indicated that rats in the vehicle group pressed more than rats in the 100 μg/kg/infusion group on days 10–20, and more than the 30 μg/kg/infusion group on day 14, while the two THC groups only differed on days 16 and 20. No significant main effects of sex or interactions emerged for active lever pressing. Similarly, there were main effects of dose [F_(2,99)_ = 3.96, p<0.05] and day [F_(19,1881)_ = 3.79, p<0.001] for inactive lever presses, as well as a significant day х dose interaction [F_(38,1881)_ = 3.04, p<0.001] with vehicle animals pressing the inactive lever more than animals in the 100 μg/kg/infusion group on days 11–12, and 16–20 and animals in the 30 μg/kg/infusion group pressing significantly more than the 100 μg/kg/infusion group on days 11 and 20. There was no significant difference in inactive lever pressing due to sex or any additional interactions (p>0.05 for all analyses). Analysis of infusions yielded main effects of day [F_(19,1881)_ = 8.34, p<0.001], and dose [F_(2,99)_ = 7.64, p<0.001] and a day х dose interaction [F_(38,1881)_ = 3.52, p<0.001]. Again, animals in the vehicle group received more infusions than rats in the 100 μg/kg/infusion group on days 10–20, and more infusions than the 30 μg/kg/infusion group on days 14,16, and 17, while the 30 μg/kg/infusion group received more infusions than the 100 μg/kg/infusion group on days 16 and 20. Thus, the greatest differences in lever pressing emerged between the vehicle and high dose groups toward the end of self-administration, when animals in the high dose group reduced their lever pressing and the number of infusions received once escalating to the highest dose available.

To investigate whether animals adjusted their intake upon escalating to the highest unit dose available, we compared the average number of infusions received on days 7–9 of training to the average number of infusions received on days 10–12 of training for all groups (Fig 1e). Both male and female rats reduced the number of infusions they chose to receive upon escalating to the 100 μg/kg/infusion dose of THC (male t_21_=2.74, p<0.05, female t_21_=2.23, p<0.05). In groups where there was no change in the unit dose available, males in the 30 μg/kg/infusion group increased their taking (t_11_=3.17, p<0.01) and females did not change (t_13_=0.78, p>0.1) over the same time period. Similarly, females in the vehicle group increased their intake (t_16_=2.77, p<0.05) while males did not show a significant increase (t_15_=1.89, p=0.078). Thus, only animals that escalated to the highest dose available showed expected titration of their intake to reflect the change in THC concentration.

To determine the total amount of THC intake, we analyzed the average daily intake for all males and females self-administering and if animals escalated intake over time (Fig 1f). A 3-way ANOVA with day of self-administration as a within subjects’ factor and sex and THC dose (30 or 100 μg/kg/infusion) as between subjects’ factors found a main effect of day [F_(19,1038)_ = 51.68, p<0.0001], a main effect of dose [F_(1, 57)_ = 30.63, p<0.0001] and a day х dose interaction [F_(19, 1083)_ = 15.16, p<0.0001] where intake in the 100 μg/kg/infusion group was higher than the 30 μg/kg/infusion group from days 10–20. There were no statistically significant effects of sex on intake. Of rats in the 100 μg/kg/infusion group, males took a mean of 1.09 ± 0.037 mg/kg and females took 1.13 ± 0.032 mg/kg. In the 30 μg/kg/infusion group, males took 0.41 ± 0.013 mg/kg and females took 0.45 ± 0.022 mg/kg.

**Fig. 2:**
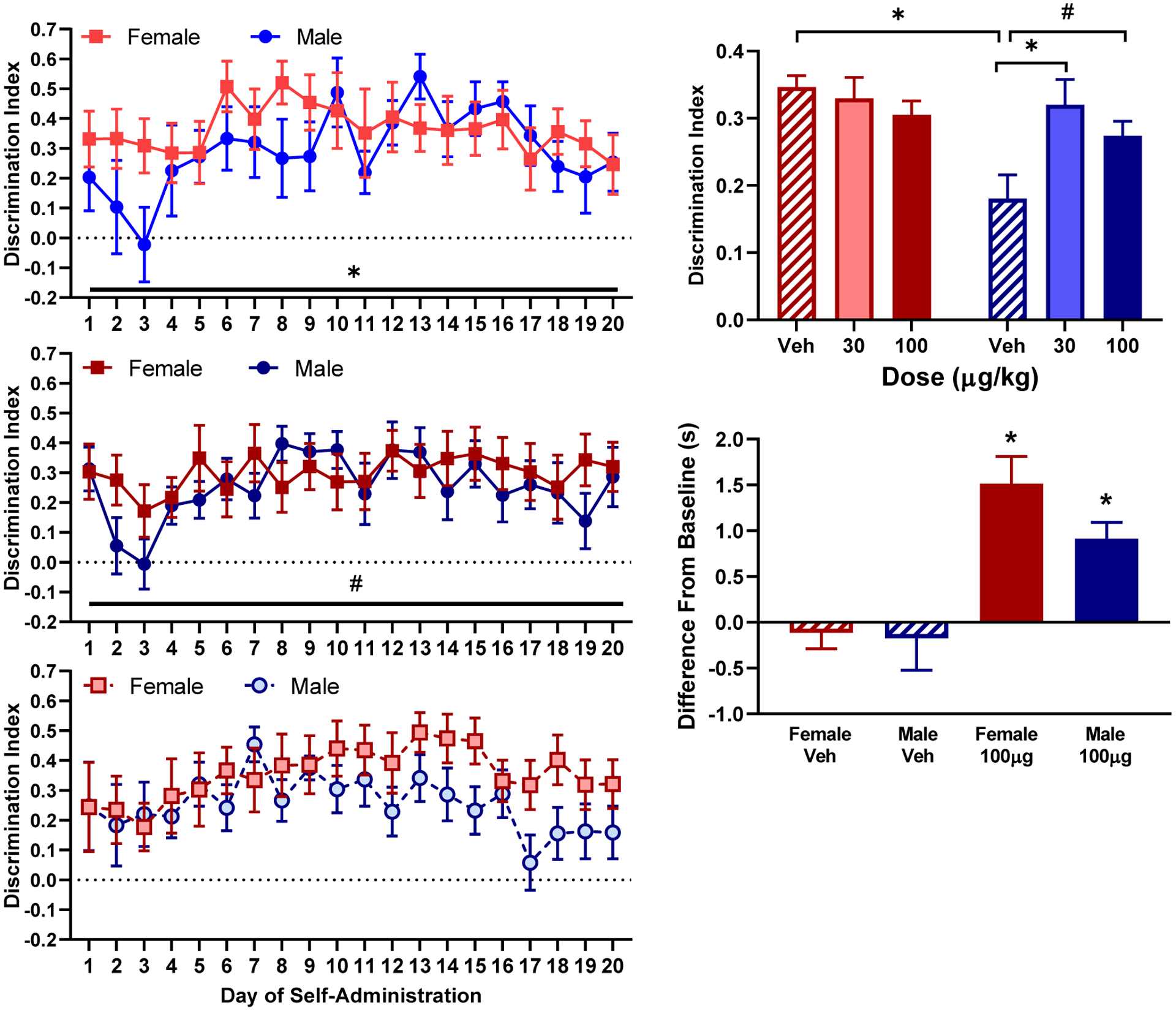
Changes in lever discrimination and antinociception throughout adolescent self-administration. (a-c) Discrimination index comparing differentiation between the active and inactive lever in all groups over 20 days of self-administration, *main effect of day, p<0.05. (d) Discrimination index comparing active and inactive lever pressing in each group from days 10–20 of self-administration, * p<0.05, # p<0.1. (e) Antinociception on the hot water tail withdrawal task in rats taking 100 μg/kg or vehicle, comparing latencies to remove the tail prior to self-administration and immediately after, *difference from baseline, p<0.05

We next evaluated the extent to which animals differentiated between active and inactive levers by computing a discrimination index for each day of self-administration. We found that male and female animals in the 30 μg/kg/infusion group increased their preference for the active lever throughout the course of self-administration (Fig 2a) with a main effect of day [F_(19,456)_ = 1.93, p<0.05] but no main effect of group or group × day interaction (p>0.05). Similarly, for animals in the 100 μg/kg/infusion group (Fig 2b) there was a trend toward a main effect of day [F_(19, 798)_ = 1.71, p=0.083] but no main effect of group or group by day interaction (p>0.05). No main effects or interactions emerged for animals self-administering vehicle (Fig 2c, p>0.05 for all analyses). To further evaluate differentiation between active and inactive levers once the animals had reached their final training dose of THC, the discrimination index was also calculated for the last 10 days of self-administration (Fig 2d). We assessed the extent to which each group pressed the active lever more than the inactive lever by computing difference scores during this period. A 2-way ANOVA with sex and dose as between subjects’ factors yielded a main effect of sex [F_(1, 54)_ = 7.07, p<0.01] and a sex х dose interaction [F_(2, 54)_ = 4.62, p<0.05]. There was no difference in scores within females, but within males the vehicle group had a significantly lower difference score than the 30 μg/kg group and trended toward a lower difference score than the 100 μg/kg group (p=0.059). Within the vehicle group, males had a significantly lower difference score than females suggesting they differentiated between the active and inactive lever less than female animals.

### Adolescent THC self-administration produces antinociceptive effects

Immediately prior to and after the 2-hour self-administration session on days 19 or 20, a subset of animals in the vehicle (n=7 male, n=7 female) or 100 μg/kg (n=9 male, n=13 female) groups underwent a tail-flick latency test for antinociception (Fig 2e). A main effect of timepoint (F_(1, 32)_ = 13.84, p<0.001) and a timepoint х group interaction emerged [F_(3, 32)_ = 9.06, p<0.001]. Post hoc comparisons indicate that both male and female rats that self-administered THC increased latency to remove their tails from the water, while animals in the vehicle group did not change.

### Young adult rats will extinguish lever pressing and show cued reinstatement after THC self-administration

**Fig. 3:**
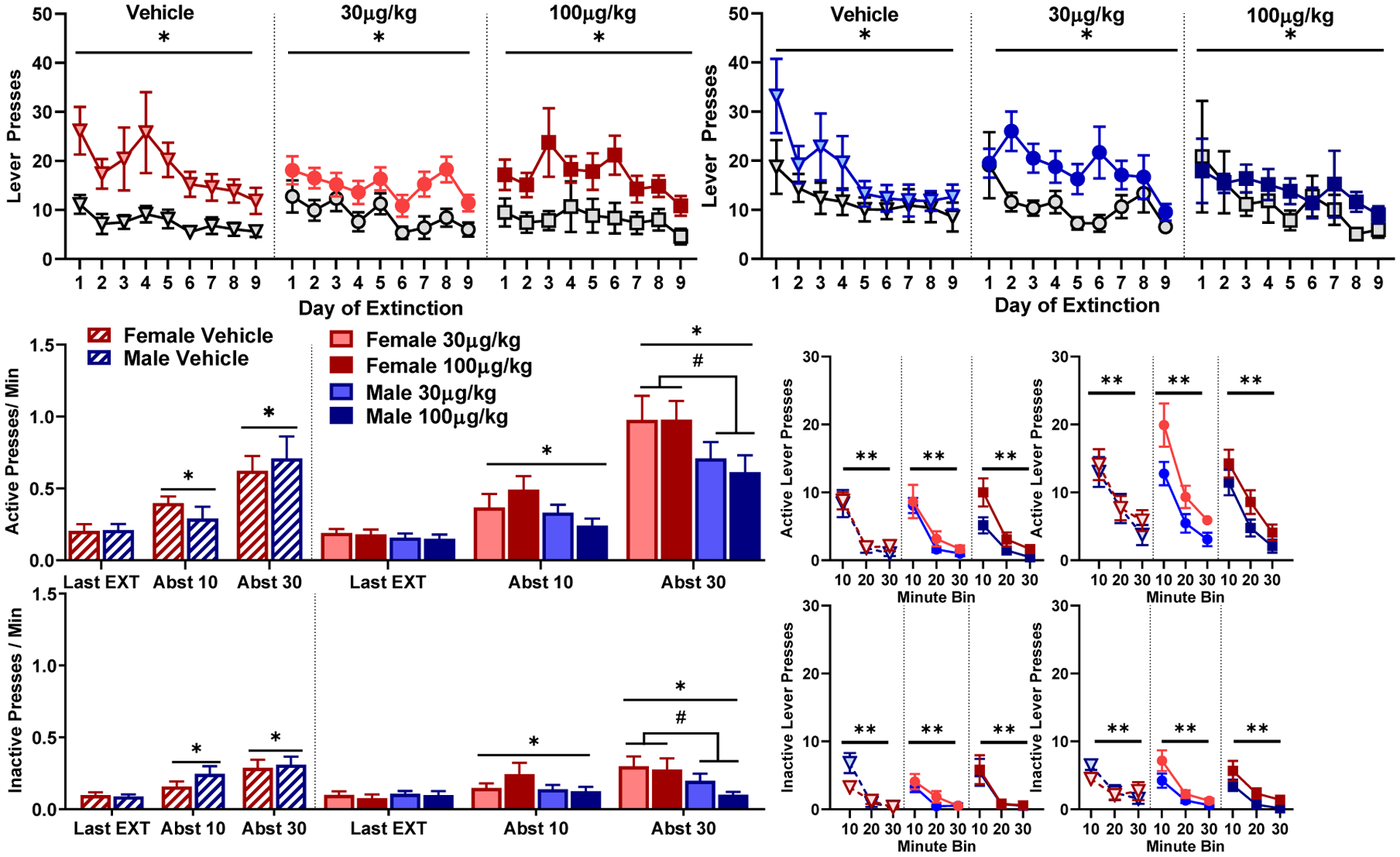
Lever extinction and cued reinstatement in rats that self-administered THC or vehicle during adolescence. (a) Lever extinction in female and (b) male rats. * main effect of day, p<0.05. (c) Active or (f) inactive lever presses per minute on the last day of extinction and abstinence days 10 and 30. * main effect of abstinence day, p<0.05, # difference between males and females, p<0.05. (d-e) Active and (g-h) inactive lever presses during the cued reinstatement sessions binned into 10 minute intervals, on (d,g) abstinence day 10 and (e,h) abstinence day 30, **p<0.01

After self-administration, male (vehicle n=11, 30 μg/kg n=12, 100 μg/kg n=13) and female (n=12 per group) rats underwent 9 days of lever extinction (PD 52–60). All rats significantly reduced lever pressing throughout extinction training (Fig 3a–b). Analysis of active and inactive lever pressing revealed a main effect of day [F_(8,536)_ = 5.11, p<0.001], [F_(8,536)_ = 5.81, p<0.001] respectively, but no other significant effects or interactions emerged.

On days 10 and 30 of abstinence (PD 61 and 81) rats underwent a test for cued reinstatement of drug-seeking (Fig 3c,f). Presses per minute were analyzed due to the difference in reinstatement and extinction session length. A sex х group х day 3-way ANOVA revealed that all groups of animals reinstated active and inactive lever pressing compared to responding during lever extinction as demonstrated by a main effect of day [active pressing: F_(2,134)_ = 88.79, p<0.001; inactive pressing: F_(2,134)_ = 25.85, p<0.001]. A main effect of sex also emerged only for active lever responding, [F_(1,67)_ = 5.11, p<0.05], where females generally increased lever pressing compared to males. Separate analyses indicate that animals in the vehicle group increased pressing over time, but there was no difference in reinstatement based on sex. To further investigate this interesting effect of sex that occurred only in the THC exposure group, a separate 3-way analysis was conducted to compare sex х THC dose х day. This analysis yielded a sex х day interaction for active [F_(2, 90)_ = 3.22, p<0.05] and inactive [F_(2, 90)_ = 4.43, p<0.05] levers, where females pressed more than males on abstinence day 30 regardless of THC dose.

Each 30 min reinstatement test session was then divided into 10-minute bins to compare lever pressing over time between groups by sex × group × time 3-way ANOVA (Fig 3d–h). Analysis of active lever pressing during the first reinstatement test session (Fig 3d), yielded main effects of sex [F_(1,68)_ = 4.94, p<0.05] and time bin [F_(2,136)_ = 81.46, p<0.001] but no sex × time interaction, suggesting that the increase in lever pressing exhibited by females was not specific to initial responding at the beginning of the session. This effect was also present in the second reinstatement test session (Fig 3e), where a main effect of sex [F_(1,68)_ = 6.25, p<0.05] and time bin [F_(2,136)_ =97.64, p<0.001] emerged. Responding on the inactive lever during the first reinstatement session (Fig 3g) also decreased over time as evident by an effect of time bin [F_(2,136)_ = 44.43, p<0.001] but there was no difference between sexes. A main effect of sex was present on the second test of reinstatement (Fig 3h) [F_(1,68)_ = 4.37, p<0.05] as well as a main effect of time bin [F_(2,136)_ = 44.59, p<0.001].

### Male rats that self-administered high doses of THC in adolescence show better working memory performance.

**Fig. 4:**
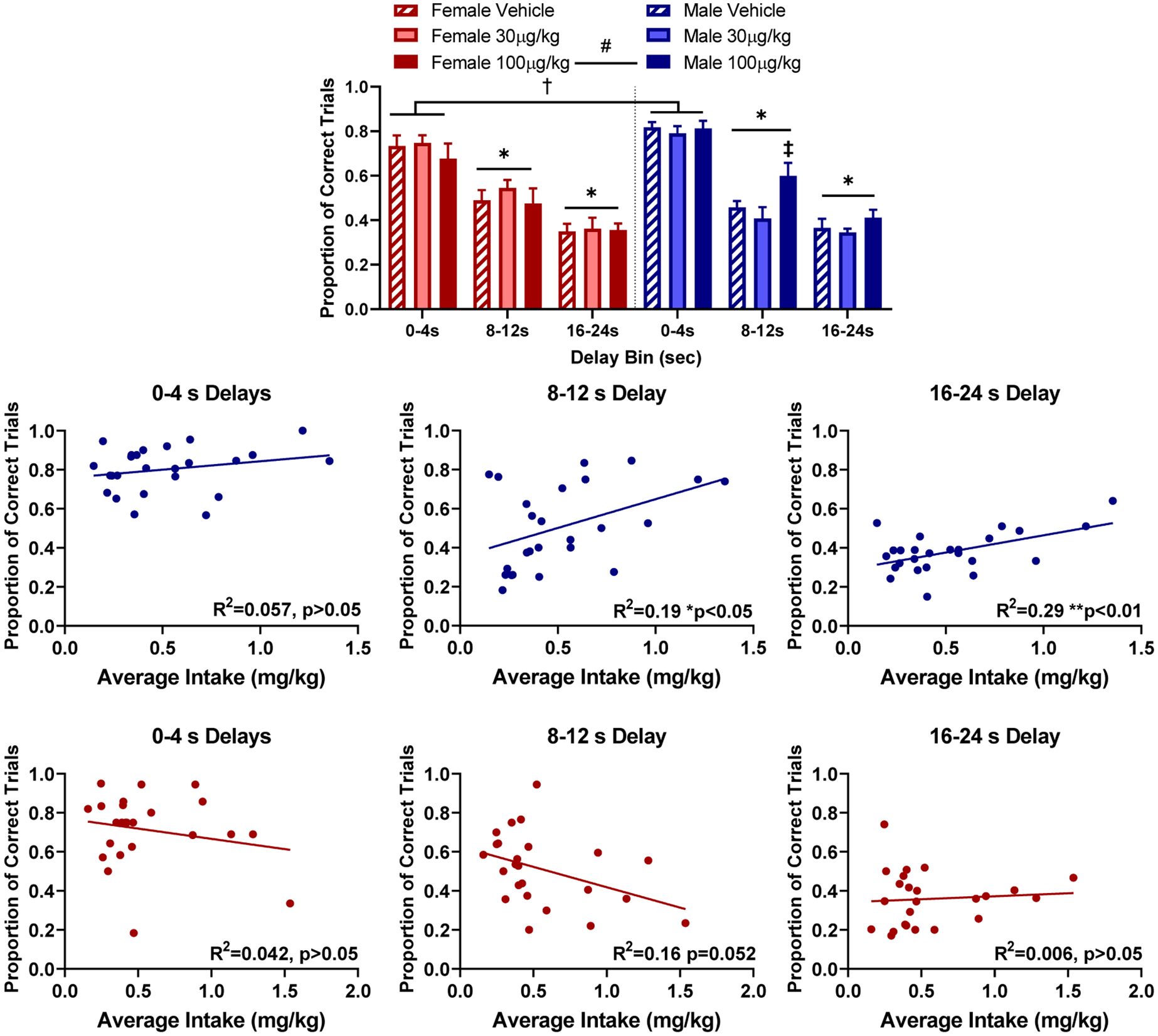
Working memory performance in adult rats that self-administered THC or vehicle during adolescence. (a) Proportion of correct trials performed during the working memory task by female (left) and male (right) rats on short (0–4s) moderate (8–12s) and long (16–24s) delays. * main effect of delay, p<0.05, # trend toward a difference in performance within rats that self-administered 100 μg/kg THC, p<0.1, † difference between males and females, p<0.05, ‡ difference between males in the 100 μg/kg group and males in the 30 μg/kg and vehicle groups, p<0.05. (b-g) Correlation between intake during the last 10 days of self-administration and the proportion of correct trials on the working memory task at the 0–4 s delays in (b) males and (e) females, the 8–12 s delays in (c) males and (f) females, and the 16–24s delay in (d) males and (g) females

Beginning at PD 63, rats that had completed self-administration and lever extinction were trained on the delayed-match-to-sample task and tested for working memory performance on delays ranging from 0.5–24s (Fig 4). Performance on each of the seven delays presented was expressed as a proportion of correct trials and binned to represent short (0.5–4s), medium (8–12s), and long (16–24s) delays (Fig 4a). These bins were chosen to compare performance on delays where animals reliably perform well (short delays, approximately 80% correct trials) to longer delays where accuracy approaches chance (33% correct trials). This method also facilitates correlations between task accuracy and THC intake during self-administration. Repeated measures analysis comparing all groups yielded a main effect of delay [F_(2,134)_ = 193.99, p<0.001], as all animals decreased in accuracy at longer delays. Additionally, there was a delay × sex interaction [F_(2,134)_ = 3.20, p<0.05] where males performed better than females at the shorter delays. There was a trend toward a sex × dose interaction [F_(2, 67)_ = 2.51, p=0.088] where male rats in the 100 μg/kg group performed better than female rats who self-administered the same dose. Further within-sex analysis of performance indicated that male animals in the 100 μg/kg group performed better than those in the vehicle and 30 μg/kg groups at moderate length delays, but not at short or long delay periods [delay × group interaction, F_(4,66)_ = 2.53, p<0.05]. Females showed no difference in performance based on THC exposure (p>0.05 for all analyses). We next correlated accuracy at each delay period with average THC intake over the last 11 days of self-administration. In male rats (Fig 4b–d), THC intake was positively correlated with increased performance at moderate delays (R^2^ = 0.19, p<0.05) and long delays (R^2^ = 0.29, p<0.01) but not short delays (R^2^ = 0.057, p>0.05). Female rats (Fig 4e–g) exhibited an opposite trend toward a decrease in accuracy with increasing THC intake. On moderate delays there was a trend toward decreased performance (R^2^ = 0.16, p=0.052) but no significant correlations emerged at other delay periods.

### Adolescent THC self-administration causes long-lasting alterations in receptor protein expression

**Fig. 5:**
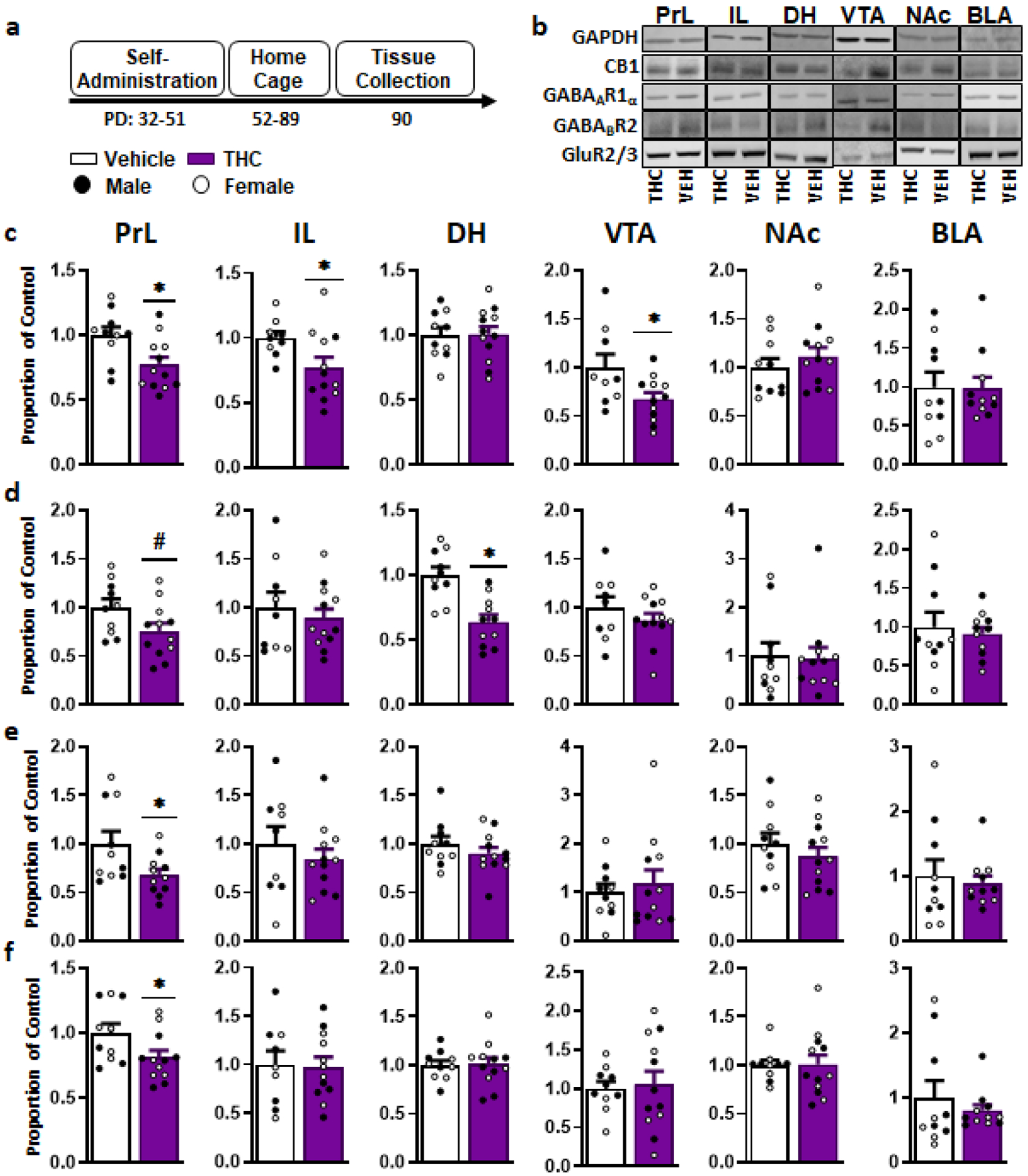
Protein expression measured in adulthood after adolescent THC or vehicle self-administration. (a) timeline of behavior and tissue collection. (b) Representative bands from THC and vehicle exposed rat tissue from the prelimbic cortex (PrL) infralimbic cortex (IL) dorsal hippocampus (DH) ventral tegmental area (VTA) nucleus accumbens (NAc) and basolateral amygdala (BLA). (Rows c-f) Protein expression shown as a proportion of immunoreactivity in the vehicle group from each of the above brain regions (n=10–12 THC samples per region, n=8–10 vehicle samples per region), females represented as open circles males represented as filled circles (c) CB1, (d) GABA_A_R1_α_, (e) GABA_B_R2, (f) GluR2/3. * Change from vehicle, p<0.05

A subset of male and female rats that completed self-administration of the high dose of THC (100 μg/kg/infusion, n=10–12) or vehicle (n=8–10) remained in the home cage after self-administration and was euthanized in adulthood at PD 90. Protein expression was analyzed by Western blot to determine if THC self-administration caused a protracted change in protein expression that persisted until adulthood (Fig 5). Male and female tissue was analyzed separately and then collapsed across sex when no sex differences were found. CB1 receptors (Fig 5c) were found to be reduced in the PrL, (t_20_= 2.67, p<0.05), VTA (t_19_=2.33, p<0.05), and IL (t_19_=2.28, p<0.05) but no other regions. Additionally, GABA_A_R1_α_ protein (Fig 5d) was found to be reduced in the DH (t_19_=4.31, p<0.001) and trended toward a reduction in the PrL (t_20_=2.04, p=0.055), while GABA_b_R2 (Fig 5e) was reduced only in the PrL (t_19_=2.31, p<0.05). GluR2/3 protein (Fig 5f) was also reduced solely in the PrL (t_20_=2.15, p<0.05). We saw no significant changes in the measured receptor proteins in the NAc or BLA.

## Discussion

Here, we demonstrate that volitional self-administration of THC can be achieved in male and female adolescent rats. This self-administration results in physiologically and behaviorally relevant exposure to the drug as indicated by acute antinociceptive effects. We identified the emergence of potential sex-specific effects in adulthood, where males exhibited enhanced working memory performance compared to females, and the greatest discrepancy in working memory performance occurred in males and females that self-administered the most drug during adolescence. Adult rats that self-administered THC in adolescence also showed reductions in multiple proteins involved in synaptic transmission, as well as cannabinoid receptors in regions of the brain that undergo developmental changes during adolescence.

This model of intravenous THC self-administration takes advantage of an escalating dose procedure, similar to those that have been used in some experimenter-administration protocols (Rubino et al. 2008), to model escalation of intake as animals develop tolerance to the effects of the drug. While intravenous self-administration of synthetic cannabinoids has been achieved in both adolescent and adult rodents at similar unit doses (Fattore et al. 2001; Lefever et al. 2014; Kirschmann et al. 2017b; Kirschmann et al. 2017a), THC self-administration in adults has only been achieved at lower doses than were reached in this study (Takahashi and Singer 1979; Wakeford et al. 2017; Spencer et al. 2018). Adolescent animals are less sensitive to the aversive effects of THC (Quinn et al. 2008), which may explain the sustained intake at higher doses than has been observed with adult self-administration. The observed differences in intake could be related to the design of the self-administration period, as the procedure used here differs from the recently published investigations of intravenous THC self-administration in adults (Wakeford et al. 2017; Spencer et al. 2018). The training period in the present study was twice as long as one study of adult self-administration (Spencer et al. 2018), and animals escalated to increasing doses instead of decreasing concentrations of THC. The escalation period used here is similar to that of another recently published study (Wakeford et al. 2017), but all rats in the present study were given daily access to THC without any days off between doses for drug washout. Additionally, we saw higher inactive lever pressing and less differentiation between active and inactive levers than has been shown during adult THC (Spencer et al. 2018) or adolescent WIN 55,212–2 self-administration (Kirschmann et al. 2017b; Kirschmann et al. 2017a). Importantly, the decrease in lever pressing upon escalation to the highest dose suggests that the THC group adjusted the amount of drug they chose to receive and had learned the association between the active lever and drug delivery.

Interestingly, we report that adolescent male and female rats in the vehicle group exhibited significant lever pressing and differentiation between the active and inactive levers. These animals also increased their lever pressing during cued reinstatement sessions. Reinstatement of THC-seeking without reinstatement of vehicle-seeking has been demonstrated in abstinent adults, primarily in animals that were exposed to both THC and cannabidiol (Spencer et al. 2018; Freels et al. 2020). The presence of cannabidiol during self-administration may account for the robust reinstatement seen in these studies. Spencer et al (2018) also reported high rates of responding for vehicle in adults which decreased over the course of training, whereas adolescents in the present study responded consistently over the course of the training period. All groups of animals in this study successfully extinguished lever pressing when neither the drug nor cue were available, and the lack of an extinction burst on the first day of extinction may be due to residual levels of circulating THC (Spencer et al. 2018). Females exhibited a particularly robust amount of responding for vehicle during self-administration and reinstatement, though both sexes increased responding compared to extinction levels during reinstatement testing. The vehicle solution used in this study has been shown to be non-reinforcing in adult animals compared to synthetic cannabinoids (Fattore et al. 2001; Lefever et al. 2014), suggesting that these adolescent animals were responding primarily for the audiovisual cue and that this cue-seeking behavior was more prominent in females. It may be that THC self-administration is closer to nicotine self-administration, another drug of abuse where rodent self-administration is difficult to achieve despite the reinforcing effects seen in humans. Cue presentations are an integral part of achieving sustainable nicotine self-administration, and rodents will perform operant responses for presentation of a visual stimulus paired with saline alone (Donny et al. 2003). The fact that adolescent animals in this study respond for the cue should not take away from the usefulness of this method to model the impact of THC delivered at self-administered doses, which are presumably not aversive, and which we found to have physiologically relevant effects.

While this model of self-administration does not produce high amounts of infusions similar to those seen with stimulants or opiates, it does produce self-administration of antinociceptive doses of THC in a range consistent with those that produce reinforcing effects in conditioned place preference and intracranial self-stimulation studies (Gardner et al. 1988; Valjent and Maldonado 2000; Braida et al. 2004; Katsidoni et al. 2013). Notably, the self-administered doses (from 0.4 mg/kg to 1.4 mg/kg THC) are lower than those used in several studies of experimenter administration that have THC-induced cognitive deficits (Cha et al. 2006; Rubino et al. 2008; Renard et al. 2017). As we do not have data on the serum or brain concentrations of THC throughout the study, our ability to draw conclusions based on intake is limited. A future serum analysis of acute and residual THC levels will allow for direct comparison with intravenous THC self-administration in adults (Spencer et al. 2018), and studies conducted with vapor or smoke self-administration models (Freels et al. 2020). Given the discrepancies in the reinforcing versus aversive properties of cannabinoids including THC, the presence of this self-administration procedure will allow further differentiation and exploration of individual differences found in animals that may be more resistant to the aversive effects of THC exposure, or who may show a preference for lower doses.

In the present study adolescent self-administration of THC did not produce cognitive deficits. In fact, working memory performance in males was similar to performance after adolescent self-administration of the synthetic cannabinoid WIN 55212–2 (Kirschmann et al. 2017b), and is similar to improvements in recognition memory or working memory reported after cannabis smoke exposure (Blaes et al. 2019; Bruijnzeel et al. 2019). The analysis in this study was designed to compare behavior in animals that self-administered both low and high doses of THC. As high-dose exposure may produce behaviorally relevant effects similar to those seen in humans, future studies can focus specifically on animals that self-administered the greatest amount of THC. While we saw no sex differences in receptor protein expression, a sex-difference may exist in the desensitization or binding of CB1 receptors that contributes the efficiency of cannabinoid receptors during memory processing. Low doses of cannabinoids can stimulate object recognition and working memory performance in aged rodent populations (Marchalant et al. 2008; Sarne et al. 2018), and it may be that the doses produced in this study interact with the developing adolescent endocannabinoid system to ultimately promote cognitive performance in adult males. Given the requirement for activity within the mPFC for working memory performance (Horst and Laubach 2009; Kirschmann et al. 2017b), the reduction of CB1 receptors in this region represents a clear area for further study.

Analysis of protein expression by Western blot returned no significant effects of sex, but uncovered reductions in key receptor proteins in the mesocorticolimbic system. CB1 receptor reductions, desensitization, or downregulation are a common effect of adolescent THC exposure at higher experimenter administered doses (Burston et al. 2010; Weed et al. 2016), and we report a similar effect in this study. Disruptions in GABA transmission have been reported after adolescent cannabinoid exposure (Cass et al. 2014; Renard et al. 2017) as well as the potential for a female specific sex-difference (Zamberletti et al. 2014). The effects seen in the current study concur with others describing altered and depressed functioning of the GABAergic, glutamatergic, and endocannabinoid systems after chronic adolescent exposure (Ellgren et al. 2008; Renard et al. 2018) and further support the ability of self-administration to produce long-term physiological effects in key brain regions associated with cognitive processing and addiction-related behaviors.

This study was designed to facilitate comparisons between adolescent THC self-administration relative to our previous work with adolescent WIN self-administration (Kirschmann et al. 2017b; Kirschmann et al. 2017a). One limitation of this approach is that behavioral experiments were performed during the light phase. As rodents are more active during the dark cycle, the timing of behavioral testing could contribute to the measured differences in performance. Future studies can be conducted during the dark phase to question if time of day contributes to THC self-administration and behavioral correlates. The animals in this study were also individually housed after surgery to protect the integrity of the indwelling catheters. Social isolation may contribute to the degree of drug-seeking, and future studies can also investigate if this isolation contributed to increased cue-seeking, particularly in the vehicle group. Our ability to discuss the cause of the observed sex-differences is limited as we did not measure estrous cycle during this study. However, in our previous study of WIN self-administration, we found that estrous cycle did not influence lever pressing during self-administration or working memory performance in adulthood (Kirschmann et al. 2017a). Similarly, a recent study in THC-exposed adult females reports that estrous cycle does not influence working memory performance on a comparable operant-based and PFC-dependent task that measures performance at the same delays tested here (Blaes et al. 2019). As estradiol has been shown to contribute to CB1 receptor expression and activity (Riebe et al. 2010; Winsauer et al. 2011; Castelli et al. 2014), future studies can be designed to specifically measure not only estrous cycle, but estradiol and other sex hormones to truly uncover how THC and circulating hormones may interact to alter receptor-level changes and behavioral performance.

## Conclusions

In conclusion, we present a rodent model for adolescent intravenous self-administration of THC and the extent of some behavioral and molecular alterations that accompany exposure to self-administered doses of the drug. Human literature reporting the effects of cannabinoid exposure during adolescence has been varied, suggesting that the magnitude of differences depends on age of onset, dose, and length of exposure (Stringfield and Torregrossa 2021). Our data suggest that self-administered doses of THC may provide similar results based on timing of exposure and dose used, and that this exposure may produce long-lasting behavioral and neuronal alterations. Future experiments can take advantage of novel models that allow for volitional control of THC exposure, to further characterize the effects of cannabinoid exposure at key developmental timepoints and produce translationally relevant comparisons.
